# Influence of *Cyperus esculentus* tubers (Tiger Nut) on male rat copulatory behavior

**DOI:** 10.1186/s12906-015-0851-9

**Published:** 2015-09-23

**Authors:** Mohammed Z. Allouh, Haytham M. Daradka, Jamaledin H. Abu Ghaida

**Affiliations:** Department of Anatomy, Faculty of Medicine, Jordan University of Science & Technology, P. O. Box: 3030, Irbid, 22110 Jordan; Biology Department, Faculty of Science, Taibah University, Al-Madinah Al-Munawwarah, 41321 Saudi Arabia

**Keywords:** *Cyperus esculentus*, Earth almond, Libido, Sexual behavior, Testosterone

## Abstract

**Background:**

*Cyperus esculentus* tubers (tiger nut) are one of the ancient food sources known to humanity. It is traditionally used in the Middle East to stimulate sexual arousal in men. However, there has been no scientific evidence about its assumed aphrodisiac properties. This study aimed to investigate the influence of tiger nut on the copulatory behavior of sexually active male rats.

**Methods:**

Two sets of sexually active male rats -highly active and moderately active- were identified depending on baseline sexual activity. Rats in each set were randomly divided into a control and treated groups. Highly active rats were treated with doses of 1 and 2 g/kg/d of raw tiger nut powder, while moderately active rats were treated with a dose of 2 g/kg/d. After 30 days’ treatment, copulatory behavior and serum hormonal levels were measured and compared between the groups within each experimental set. Phytochemical analyses including liquid chromatography/mass spectrometry and atomic absorption were performed to elucidate the main constituents of tiger nut that may be responsible for altering serum hormones.

**Results:**

Tiger nut stimulated sexual motivation in both highly and moderately active rats, indicated by reduced mount and intromission latencies in these rats compared to controls. Furthermore, tiger nut improved sexual performance, indicated by increased intromission frequency and ratio, in treated moderately active rats compared to controls. Serum testosterone levels increased significantly after tiger nut administration. Lastly, phytochemical analyses revealed the presence of quercetin, vitamin C, vitamin E, and mineral zinc in tiger nut.

**Conclusions:**

Tiger nut has positive effects on the copulatory behavior of adult male rats.

## Background

*Cyperus esculentus* L. varsativus (*C. esculentus*) is a perennial plant species that belongs to the Cyperaceae family and grows abundantly in the Mediterranean region [1, 2]. Tubers of this plant are considered one of the earliest food sources known to humanity, where they have been documented to be cultivated by ancient Egyptians since 5000 BC [1, 3]. These tubers are commonly known by several names such as chufa, earth almond, and tiger nut [1, 2]. *C. esculentus* is a potentially valuable food source for humans and animals due to its rich nutritional contents of fat, carbohydrates, and minerals [4]. However, it is very important to differentiate between this species and its variant wild type weed plant known as yellow nutsedge, which is native to North America [1, 2].

In addition to being a food source, *C. esculentus* tubers have several other purposes. For example, in Spain, they are used in the preparation of a milk-like beverage named “horchata” [2]. The milk concentrate of the tubers is also used in the manufacturing of some cosmetic products [5]. Furthermore, pork burgers containing liquid co-products from *C. esculentus* tubers have demonstrated improved cooking properties compared to those without [6].

According to Ayurvedic medicine, *C. esculentus* tubers can be used for their aphrodisiac properties [7]. In the Middle East, they are known to the public as “Hab Al-zulom” (Arabic), which translates to “the seeds of men”, owing to their apparent ability to improve male sexual activity; thus, they are frequently given to grooms during their honeymoons as a sexual invigorator. However, there has been no scientific evidence to date on the influence of *C. esculentus* tubers on male sexual behavior.

In a previous study, Al-Shaikh et al. [8] reported protective effects of *C. esculentus* on testicular weight and spermatogenesis process in mice treated with lead acetate. They speculated that these effects could be due to either the antioxidant ability of *C. esculentus* or its positive influence on sex hormones. In addition, it has been claimed that treatment with *C. esculentus* methanolic extract improves sperm count and motility in male rats, which is associated with increased gonadotropins and testosterone serum levels [9].

Based on the previous facts, this study aimed to investigate the effects of *C. esculentus* tubers (tiger nut) on the copulatory behavior of adult male rats. In addition, serum hormonal assays and phytochemical analyses were conducted to elucidate the mechanism by which tiger nut may influence sexual behavior.

## Methods

### Experimental model

Adult male Sprague-Dawley rats (weight: approx. 250 g) raised in the Animal House Unit at Jordan University of Science and Technology (JUST) were used in this study. Animal care and experimental procedures were approved by the Animal Care and Use Committee at JUST and were in accordance with the NIH Guidelines. The animals were maintained under controlled temperature (21 ± 1 °C) with a 12 h light and 12 h darkness schedule (lights on, 06:00–18:00 h). Food and water were provided *ad libitum*. The rats were acclimatized for two weeks before beginning the experiments.

Male rats were subjected to four pre-experimental mating tests to acquire sexual experience. After that, additional three mating tests were conducted with sexually receptive females to determine the baseline sexual activity of the rats. Male rats that achieved ejaculation in each of the three tests in less than 30 min were considered highly active, those that achieved ejaculation in one or two of the three tests in less than 30 min were considered moderately active, and those that failed to achieve ejaculation in any of the three tests in less than 30 min were considered sexually inactive and excluded from the study [10]. Ejaculation was monitored by rhythmic contractions of the posterior abdomen that ended with slow raising of the forelimbs [11].

Highly active rats were randomly divided into three groups (*n* = 16 rats/group): a control (C) group that received distilled water; Tiger Nut 1 (T1), treated with 1 g/kg/d tiger nut; and Tiger Nut 2(T2), treated with 2 g/kg/d of tiger nut. Moderately active rats were randomly divided into two groups (*n* = 16 rats/group): a control (C) group that received distilled water, and a treated (T) group treated with 2 g/kg/d tiger nut. These doses are considered comparable with the human dose, since an average human male usually consumes between 100 and 200 g daily of dried tiger nut.

Female rats of the same strain were also used in this study. Each female was brought into estrus by sequential subcutaneous injections of 50 μg estradiol benzoate (Intervet International B.V., Holland) and 1 mg progesterone (Schering AG, Germany) 48 and 4 h before the mating tests, respectively. The females were screened with non-experimental males, and the ones that showed good sexual receptivity (solicitation and lordosis) were selected for the behavioral test.

### Treatment

**Fig. 1:**
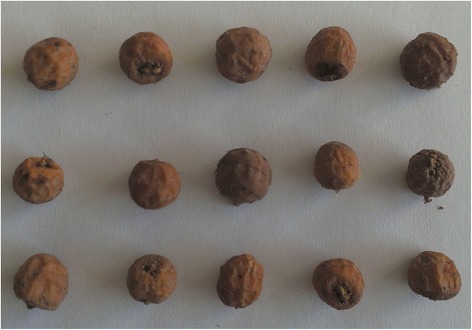
*Cyperus esculentus* L. var sativus tubers (tiger nut)

Underground tubers of *C. esculentus* were harvested from a region in north Jordan near the ancient biblical city of Gerasa (32°16′37″N, 35°53′25″E). The harvested tubers were further identified by specialized botanists in the department of biological sciences at Yarmouk University, Jordan. A voucher specimen of the tubers (NHJ.2014.0001) was deposited in the National Herbarium of Jordan at the Royal Botanic Garden of Jordan (Fig. 1). The tubers were air dried and ground into powder to facilitate their administration to the animals. Tiger nut powder was administered to the rats daily for 30 days by oral gavage to avoid the fluctuations in the rate of testosterone secretion [12]. The assigned dose of the powder was suspended in 2 ml of distilled water before being administered to the animals. The control groups received a daily dose of 2 ml distilled water.

### Copulatory behavior test

The copulatory behavior of male rats was monitored by two trained observers blinded to the experimental design, in a sound-attenuated room. Ten male rats from each group were subjected to the sexual behavior test. The test was performed 24 h after the last treatment and during the dark phase of the light/dark cycle. A single male rat was placed in a rectangular Plexiglas observation chamber (45 × 40 × 30 cm) and allowed to acclimate for 5 min. A sexually receptive female rat was then introduced into the chamber. The following parameters of sexual behavior were measured as described previously [10, 13]: (1) Mount latency: time from introduction of the female until the first mount; (2) intromission latency: time from introduction of the female until the first intromission (vaginal penetration); (3) mount frequency: number of mounts preceding ejaculation; (4) intromission frequency: number of intromissions preceding ejaculation; (5) ejaculation latency: time from the first intromission until ejaculation; (6) post-ejaculatory interval: time from ejaculation until the next intromission; (7) intromission ratio: a measure of intromissive success calculated as intromission frequency/(mount frequency+intromission frequency); and (8) inter-intromission interval: the average interval between successive intromissions calculated as ejaculation latency/intromission frequency. Tests were ended immediately after the first post-ejaculatory intromission. The percentage of ejaculating rats was calculated based on the ratio of the number of rats that achieved ejaculation from the first test within a period of 30 min to the total number of rats assessed in that group.

### Body and reproductive organ weights

Total body and internal reproductive organ weights were assessed in male rats that were not submitted to mating tests (*n* = 6/group). The rats were weighed and then euthanized with ethyl ether. Subsequently, blood was obtained by cardiac puncture and collected into centrifuge tubes for later analyses. The reproductive organs (testes, epididymides, seminal vesicles, prostate, and vasa deferentia) were removed, cleaned free of fat, and weighed.

### Serum biochemistry and hormonal assays

Serum was prepared by centrifugation of the collected blood at 3000 rpm for 30 min and stored at −40 °C for later assays. Total serum protein, cholesterol, triglycerides, urea, creatinine, aspartate aminotransferase, alanine aminotransferase, creatinine kinase, and lactate dehydrogenase concentrations were determined by electrochemiluminescence immunoassay technology using appropriate assay kits (Roche Diagnostics, Mannheim, Germany). Similarly, the serum concentrations of lutropin (LH), follitropin (FSH), and testosterone were also measured.

### Phytochemical analyses

#### Liquid chromatography/mass spectrometry

Liquid chromatography coupled with tandem mass spectrometry (LC-MS/MS) was applied to identify the tiger nut constituents that may be responsible for elevating serum testosterone level. A 100 mg sample of tiger nut powder was dissolved in 4 ml methanol. The sample was vortexed for 1 min and centrifuged at 4500 rpm for 5 min, after which the supernatant was separated and evaporated at 40 °C under N_2_to dryness. The dry extract was reconstituted with 200 μl of deionized water and methanol (1:1) and then centrifuged again at 15000 rpm for 3 min. Twenty microliters of the reconstituted extract was injected into the LC-MS/MS analyzer (3200 Qtrap, AB, Canada) in the electrospray ionization positive-ion mode. Reverse-phase chromatography of the water/acetonitrile gradient program was performed for 15 min on a C18 column (Agilent Eclipse XDB; Agilent Technologies, CA, USA) to elute the extract. Proper standards were obtained and used as controls for quercetin (Sigma-Aldrich ChemieGmbh, Munich, Germany), and vitamins A (Toronto Research Chemicals, ON, Canada), C (Toronto Research Chemicals),E (Sigma-Aldrich) and B6 (Sigma-Aldrich).

#### Atomic absorption

Zinc and Selenium contents in tiger nut were determined using an atomic absorption spectrometer (AAS, Model SOLAAR M5, Unicam, UK) that was fully equipped for flame and graphite furnace atomization. Autosamplers (model FS 95) were used with the furnace to provide consistent sample introduction.

### Statistical analysis

Rats were classified into highly active and moderately active categories based on their baseline sexual activity as described earlier. Highly active rats were randomly divided into three groups (one control and two treated groups). Moderately active rats were randomly divided into two groups (control and treated). The data were evaluated by either a one-way analysis of variance (ANOVA) for the highly active category or independent sample *t*-test for the moderately active category. If a significant difference (*P* <0.05) was detected among the highly active groups, then Fisher’s least significant difference (LSD) test was performed for post hoc analysis.

## Results

### Copulatory behavior

In highly active active rats, both treated groups showed a significant (*P* <0.01) reduction in mount latency compared to the control group. In addition, there was a significant reduction in intromission latency in both T1 (*P* <0.05) and T2 (*P* <0.01) groups compared to the control group. No significant variations were found for any of the other copulatory behavior parameters between the groups of highly active male rats (Table 1). The percentage of ejaculating rats was 100 % in all groups of highly active rats.

**Table 1:** Effects of 30 days of treatment with *C. esculentus* tubers (tiger nut) on copulatory behavior parameters of highly active male rats

Treatment	ML *(s)*	MF	IL *(s)*	IF	EL *(s)*	PEI *(s)*	IR	III *(s)*
C	74.36 ± 11.25	4.36 ± 0.54	124.55 ± 14.14	11.00 ± 1.50	545.20 ± 97.41	395.70 ± 20.75	0.71 ± 0.02	42.38 ± 4.94
T1	40.75 ± 3.32^**^	4.00 ± 0.47	90.22 ± 16.10^*^	10.67 ± 0.85	374.00 ± 89.79	365.56 ± 41.79	0.73 ± 0.01	34.32 ± 6.42
T2	35.57 ± 2.97^**^	3.40 ± 0.43	54.00 ± 6.58^**^	11.60 ± 1.51	307.40 ± 23.15	330.90 ± 12.76	0.76 ± 0.02	29.71 ± 3.70

Values are means ± SEM obtained for 10 rats per group

*ML* Mount latency, *MF* Mount frequency, *IL* Intromission latency, *IF* Intromission frequency, *EL* Ejaculation latency, *PEI* Post-ejaculatory interval, *IR* Intromission ratio, *III* Inter-intromission interval, *C* Control rats treated with distilled water, *T1* Rats treated with 1 g/kg of tiger nut/d, *T2* Rats treated with 2 g/kg of tiger nut/d

^*^ *P* <0.05 from the control

^**^ *P* <0.01 from control (ANOVA)

In moderately active rats, the treated group had significantly (*P* <0.01) shorter mount and intromission latency periods than that observed in the control group. The treated group had significantly (*P* <0.01) greater intromission frequency compared to the control group. Furthermore, the treated group exhibited a significant reduction in ejaculation latency (*P* <0.01) and post-ejaculatory interval (*P* <0.05) compared to the control group. The increase in intromission frequency and reduction in ejaculation latency in the treated group were also reflected in a significantly greater (*P* <0.01) intromission ratio and shorter (*P* <0.05) inter-intromission interval compared to the control group (Table 2). The percentage of ejaculating rats increased significantly (*P* <0.05) from 50 % in the control group to 90 % in the treated group.

**Table 2:** Effects of 30 days of treatment with *C. esculentus* tubers (tiger nut) on copulatory behavior parameters of moderately active male rats

Treatment	ML *(s)*	MF	IL *(s)*	IF	EL *(s)*	PEI *(s)*	IR	III *(s)*
C	111.45 ± 11.97	4.92 ± 0.43	192.50 ± 21.43	8.42 ± 0.87	878.67 ± 85.38	432.89 ± 20.80	0.63 ± 0.01	114.94 ± 16.53
T	52.30 ± 3.89^**^	4.10 ± 0.31	92.80 ± 8.69^**^	13.10 ± 0.72^**^	470.90 ± 81.69^**^	365.50 ± 16.13^*^	0.76 ± 0.01^**^	37.35 ± 7.34^**^

Values are means ± SEM obtained for 10 rats per group

*ML* Mount latency, *MF* Mount frequency, *IL* Intromission latency, *IF* Intromission frequency, *EL* Ejaculation latency, *PEI* Post-ejaculatory interval, *IR* Intromission ratio, *III* Inter-intromission interval, *C* Control rats treated with distilled water, *T* Rats treated with 2 g/kg of tiger nut/d

^*^ *P* <0.05

^**^ *P* <0.01 (*t*-test)

### Body and internal reproductive organ weights

There were no significant (*P* >0.05) differences in body weight between control and treated rats in both highly active and moderately active categories. Similarly, the relative weights of reproductive organs to body weight were comparable (*P* >0.05) between control and treated rats in both categories.

### Serum biochemical and hormonal levels

All serum biochemicals investigated were comparable across all groups and within normal values reported by Alemán et al. [14]. In addition, there were no significant (*P* >0.05) differences in LH and FSH levels between control and treated rats in both highly active and moderately active groups (Tables 3 and 4). However, serum testosterone levels were significantly higher in highly active rats in both the T1 (*P* <0.05) and T2 (*P* <0.01) groups compared to the control group (Table 3). Similarly, a significant (*P* <0.01) increase in serum testosterone level was found in moderately active rats in the treated group compared to those in the control group (Table 4).

**Table 3:** Serum hormonal levels in highly active male rats after 30 days of treatment with *Cyperus esculentus* tubers (tiger nut)

Group	LH (IU/L)	FSH (IU/L)	Testosterone (ng/dl)
C	2.4 ± 0.3	2.1 ± 0.3	1.8 ± 0.2
T1	2.0 ± 0.3	1.6 ± 0.3	3.0 ± 0.3*
T2	1.7 ± 0.2	1.4 ± 0.3	3.4 ± 0.4**

Values represent the means ± SEM for three different groups (*n* = 6/group)

*C* Control group that received a daily dose of 2 ml distilled water, *T1* Group treated with 1 g/kg/d tiger nut powder, *T2* Group treated with 2 g/kg/d of tiger nut powder, *LH* Lutropin, *FSH* Follitropin

*P <0.05

**P <0.01 compared to control (ANOVA, Fisher’s *post hoc*)

**Table 4:** Serum hormonal levels in moderately active male rats after 30 days of treatment with *Cyperus esculentus* tubers (tiger nut)

Group	LH (IU/L)	FSH (IU/L)	Testosterone (ng/dl)
C	2.2 ± 0.3	2.1 ± 0.3	1.2 ± 0.2
T	2.0 ± 0.2	1.7 ± 0.2	2.7 ± 0.3**

Values represent the means ± SEM for two different groups (*n* = 6/group)

*C* Control group that received a daily dose of 2 ml distilled water, *T* Group treated with 2 g/kg/d of tiger nut powder, *LH* Lutropin, *FSH* Follitropin

**P <0.01 compared to control (*t*-test)

### Phytochemistry

LC-MS/MS analysis revealed the presence of several compounds in tiger nut that may boost serum testosterone levels and contribute to the improvement of the copulatory behavior: quercetin, vitamin E, and vitamin C. The concentrations of these compounds in tiger nut powder are shown in Table 5. In addition, atomic absorption spectroscopy revealed the presence of the mineral zinc in tiger nut at a concentration of 31 mg/kg. No selenium was detected in our tiger nut sample.

**Table 5:** Constituents identified in *C. esculentus* tubers (tiger nut) by liquid chromatography/mass spectrometry that are expected to contribute to higher levels of serum testosterone and to the improvement of copulatory behavior

Compound	Concentration (mg/kg)
Quercetin	10.3
Vitamin E	460.6
Vitamin C	1460.0

## Discussion

To our knowledge, this is the first study to investigate the influence of *C. esculentus* tubers, known as tiger nut, on the copulatory behavior of adult male rats. Tiger nut was found to enhance sexual motivation (desire) in highly active and moderately active male rats, and to improve the sexual performance (potency) in moderately active rats. This was accompanied by an upsurge in total serum testosterone concentration in treated rats in both categories.

The enhancement in sexual arousal in highly active male rats was evidenced by reduced mount and intromission latencies in the tiger nut-treated groups (T1 and T2) compared to the control group. Mount and intromission latencies are measurements of, and inversely proportional to, sexual motivation [15]. However, the lack of difference in other copulatory parameters suggests that there was no improvement in sexual performance in these animals.

Reduced mount and intromission latencies indicate that tiger nut treatment also stimulated sexual desire in moderately active rats. In addition, an improvement in sexual performance was evidenced in these rats through increased intromission frequency and ratio parameters after tiger nut administration. Intromission frequency reflects the efficient activation of ejaculatory reflexes, while intromission ratio, also known as copulatory efficacy or hit rate, represents the facilitation of erection and penile orientation [10, 13]. Both parameters are considered pure measurements of sexual performance in male rats. This improvement in sexual performance was further corroborated by the increased percentage of ejaculating rats in the treated moderately active group compared to the control group.

The enhanced sexual behavior in male rats after treatment with tiger nut could be attributed, in part, to the significant increase in serum testosterone levels observed in the treated animals compared to the controls. However, the role of testosterone in improving sexual behavior is still under debate. Some researchers suggest that testosterone has little, if any, impact on sexual activity [16–18]. For example, Damassa et al. [19] reported no correlation between circulating testosterone levels and male sexual behavior in normal active rats. Antonio-Cabrera and Paredes [17] reported that chronic treatment with testosterone did not improve the copulatory behavior of sexually sluggish male rats. Furthermore, a meta-analysis reported that testosterone has only small to moderate effects on sexual function in men [16]. However, the lack of statistical clarity and the way in which different study designs were combined in this meta-analysis raises the possibility of bias.

Other researchers support the idea that sexual desire and potency are dependent on testosterone levels in the blood [20, 21]. It has been claimed that a slight elevation in testosterone level can lead to a significant increase in sexual desire in men [22, 23]. Anderson et al. [24] reported that administering supra-physiological doses of testosterone enanthate can stimulate sexual arousal, but not activity, in normal eugonadal men. This supports our results on sexually highly active rats, where the increase in testosterone after tiger nut treatment coincided with enhanced sexual motivation but not performance in these animals. In a recent meta-analysis, testosterone supplementation was found to significantly improve erectile function and libido in hypogonadal men with low testosterone levels [25]. Our findings correspond with those of this meta-analysis, where an increase in testosterone level after tiger nut consumption coincided with improved sexual motivation and performance in moderately active male rats.

The exact mechanism by which tiger nut boosts testosterone levels is not entirely clear. However, we speculate that tiger nut may act directly on testicular cells, and not through the hypothalamus-pituitary axis, since no variations in FSH and LH levels were observed due to tiger nut treatment. The phytochemical analyses revealed the presence of several components (quercetin, vitamins E and C, and the mineral zinc) in tiger nut that could positively contribute to testosterone production and improve the erectile function.

Quercetin is a dietary flavonoid that exhibits strong antioxidant activity. A previous study revealed that oral administration of quercetin was associated with a significant increase in serum testosterone level in male rats [26]. Taepongsorat et al. [27] reported that subcutaneous injections of quercetin over a period of 1 week significantly increased testis weight and improved sperm quality in rats. Zhang et al. [28] suggested that quercetin could ameliorate erectile dysfunction in diabetic rats by inhibiting oxidative stress. Moreover, a recent study revealed that quercetin treatment can improve the arterial erectile dysfunction by up-regulating intracavernous pressure in Wistar rats [29].

Vitamin E is an essential nutrient speculated to enhance testosterone synthesis and was found to increase serum testosterone in an experimental aged mice model [30]. Salama et al. [31] reported that daily oral administration of 40 mg/kg vitamin E for 3 weeks restored serum testosterone concentration to normal value in an atherosclerotic rat model. The daily supplements of vitamin E in a dose of 75 mg/kg for 50 days ameliorated serum testosterone levels in rats exposed to noise stress [32]. Furthermore, Helmy and Senbel [33] reported that antioxidant therapy with vitamin E ameliorates the age-associated erectile dysfunction in male rats. In a recent study, Kawakami et al. [34] reported that vitamin E therapy can improve the poor semen quality and increase plasma testosterone levels in dogs.

Vitamin C is a strong antioxidant that facilitates the formation of testosterone, and was found in a considerable concentration in tiger nut in this study. It has been reported that vitamin C increases testosterone content in rat testis *in vitro* [35], and oral administration of vitamin C for 10 weeks significantly increased serum testosterone levels in male rats [36]. Moreover, vitamin C supplementation attenuated testosterone deficiency and some male reproductive deficits induced in hyperglycemic rats [37, 38]. Vitamin C, also, stimulates vascular nitric oxide production, which consequently improves the erectile function [39].

The trace element zinc is speculated to play a critical role in sexual development. Zinc deficiency was found to disrupt testicular tissue [40], impair spermatogenesis [41], and reduce testosterone levels [42], while zinc supplementation improved the sexual behavior of adult male rats in a dose-dependent manner by enhancing testosterone secretion [43]. Moreover, Prasad et al. [44] reported a positive correlation between cellular zinc concentration and serum testosterone level in healthy men.

## Conclusions

The present study supports the hypothesis that *C. esculentus* tubers have aphrodisiac activity, enhancing male sexual libido and performance. The observed improvements in copulatory behavior after the administration of tiger nut could be partially attributed to increased serum testosterone levels in male rats. This increase in testosterone level is most likely related to the presence of quercetin, vitamins, and zinc in tiger nut, all of which have been shown to boost testosterone production. Nevertheless, future investigations are warranted to confirm these effects of tiger nut in humans, and to examine the safety of these tubers on developing boys, since they are involved in the food and beverage industry.
